# Evaluation of Intestinal Barrier Biomarkers and Glucagon-like Peptide-2 in SIRS-Positive and SIRS-Negative Diarrheic Calves

**DOI:** 10.3390/ani16162607

**Published:** 2026-08-20

**Authors:** Adem Şahan, Sercan Hüseyin Bayendur, Canberk Balıkçı, Ahmet Cihat Tunç, Abuzer Acar

**Affiliations:** 1 Department of Internal Medicine, Faculty of Veterinary Medicine, Harran University, Şanlıurfa 63200, Turkey; ademsahan@harran.edu.tr (A.Ş.); canberkbalikci@gmail.com (C.B.); 2 Department of Internal Medicine, Faculty of Veterinary Medicine, Afyon Kocatepe University, Afyonkarahisar 03200, Turkey; cihat.tunc@gmail.com

**Keywords:** glucagon-like peptide-2, intestinal barrier, neonatal calf diarrhea, systemic inflammatory response syndrome, zonula occludens-1

## Abstract

This study investigated circulating tight junction (TJ) proteins (Zonula Occludens-1 [ZO-1], Occludin, Claudin-3) and Glucagon-Like Peptide-2 (GLP-2) concentrations across varying clinical severities of neonatal calf diarrhea. Thirty-six calves—categorized by Systemic Inflammatory Response Syndrome (SIRS) criteria into SIRS-positive (*n* = 12), SIRS-negative (*n* = 11), and healthy control (*n* = 13) groups—were evaluated via ELISA. In SIRS-negative calves, serum ZO-1 levels were significantly lower than in healthy controls (*p* = 0.0276) and positively correlated with leukocyte counts (*r* = 0.64). Conversely, SIRS-positive calves exhibited significantly higher GLP-2 levels compared with SIRS-negative calves (*p* = 0.0040), whereas serum ZO-1 levels did not differ significantly from healthy controls. Significant positive correlations (|*r*| > 0.50, *p* < 0.05) were identified between GLP-2 and TJ proteins within the SIRS-positive cohort. In conclusion, circulating GLP-2 and ZO-1 may serve as potential candidate biomarkers for monitoring intestinal barrier profiles and clinical severity in diarrheic calves, though further validation in larger prospective cohorts is warranted.

## Simple Summary

Neonatal calf diarrhea is a severe condition that can rapidly progress from a localized enteric infection to a life-threatening systemic illness. During disease progression, intestinal barrier breakdown allows harmful bacteria to enter the bloodstream. This study aimed to determine how tight junction proteins—which seal the intestinal epithelium—and glucagon-like peptide-2, a gut-trophic repair hormone, respond as clinical severity worsens. Thirty-six calves were evaluated. Early-stage diarrhea was characterized by marked intestinal barrier disruption. However, as the illness progressed to severe clinical stages, the intestinal barrier did not undergo continuous uncompensated breakdown. Instead, elevated circulating levels of GLP-2 coordinated with tight junction proteins, suggesting a potential compensatory physiological response. These findings indicate that the calf’s body exhibits concurrent biomarker alterations that may be associated with mucosal defense mechanisms even during severe clinical states. Understanding this natural defense mechanism provides valuable insights for developing improved diagnostic tools and targeted therapies, ultimately helping to lower mortality, enhance animal welfare, and mitigate economic losses in cattle production.

## 1. Introduction

Neonatal calf diarrhea remains a major health challenge in cattle production, driving high morbidity, mortality, and substantial economic losses [1,2]. Rather than remaining localized within the enteric mucosa, diarrheal infections can rapidly escalate into critical systemic illness via the translocation of pathogens and toxins into the circulation [3]. As disease progresses, clinical deterioration may culminate in systemic inflammatory response syndrome (SIRS), bacteremia, and septic shock [4,5]. This systemic involvement—characterized by severe acidemia, hypoglycemia, hypernatremia, tachypnea, and coma—markedly increases mortality risk in affected calves [6].

Under physiological conditions, the intestinal epithelial barrier serves as the primary defense against the translocation of luminal enteropathogens and endotoxins into systemic circulation. Selective mucosal permeability is coordinated by tight junction proteins, primarily members of the Zonula Occludens-1 (ZO-1), occludin, and claudin families [7]. Common enteropathogens in diarrheic calves—such as *Escherichia coli* and *Clostridium perfringens*—and their secreted toxins directly target epithelial cells, disrupting TJ architecture and triggering their dissociation from the cell membrane [8]. During localized infection, the down-regulation of key tight junction proteins (e.g., ZO-1, occludin, claudin-1) compromises barrier integrity. This hyperpermeability induces passive tissue injury and facilitates systemic toxin leakage, ultimately driving robust pro-inflammatory cascades both locally and systemically [9,10].

Glucagon-Like Peptide-2 (GLP-2) plays a pivotal role in mucosal repair and the mitigation of pathogen-induced damage. Secreted by enteroendocrine L cells, GLP-2 acts as an intestine-specific trophic factor that promotes enterocyte proliferation, inhibits apoptosis, and upregulates the resynthesis of disrupted tight junction proteins [11,12]. Evidence from livestock models demonstrates that GLP-2 enhances mucosal adaptation and substantially attenuates enteropathogen-induced intestinal injury [13,14]. Particularly under conditions such as severe systemic inflammation or barrier breakdown, evidence suggests that the organism may mount a compensatory response mediated by GLP-2 to restore intestinal integrity and modulate its physiological interaction with the immune system [11]; however, direct evidence confirming this specific mechanism during neonatal calf sepsis remains limited.

Although several studies have investigated sepsis and systemic inflammatory response syndrome (SIRS) in neonatal calf diarrhea using various biomarkers, clinical studies simultaneously evaluating the profiles of the intestinal barrier proteins ZO-1, occludin, and claudin-3, alongside GLP-2, remain limited. Accordingly, the present study aims to investigate, via biomarkers, the intestinal barrier injury occurring in relation to clinical SIRS staging in diarrheic calves, as well as the compensatory physiological mechanisms mounted by the organism against this condition.

## 2. Materials and Methods

### 2.1. Ethical Approval and Study Population

All animal handling and clinical procedures were reviewed and approved by the Harran University Animal Experiments Local Ethics Committee (Date: 6 February 2026, Decision No.: 2026/001). Prior to enrollment, calf owners were fully informed regarding the study objectives and procedures, and written informed consent was obtained. The study cohort comprised 36 neonatal calves, including 23 calves with naturally occurring neonatal diarrhea and 13 healthy controls showing no clinical signs of systemic or localized illness.

### 2.2. Clinical Examination, Group Assignment and Exclusion Criteria

To detect the presence of common enteropathogens in all diarrheic calves enrolled in the study, fecal samples were analyzed using a commercial rapid antigen test kit (BoviD-5 Ag Test Kit^®^, Bionote Inc., Hwaseong, Gyeonggi, Republic of Korea). Only calves with positive antigen test results were assigned to the diseased groups. To standardize the clinical severity stages of the disease the diarrheic calves were divided into two subgroups—SIRS-positive and SIRS-negative—based on systemic inflammatory response syndrome (SIRS) criteria widely accepted in the veterinary literature.

SIRS diagnosis was established based on the concurrent presence of at least two of the following criteria: (i) body temperature < 38.5 °C or >39.5 °C; (ii) heart rate > 120 beats/min (tachycardia); (iii) respiratory rate > 36 breaths/min (tachypnea); and (iv) total leukocyte count < 5.0 × 10^3^ cells/μL or >12.0 × 10^3^ cells/μL [4,6].

- **SIRS-Positive Group (*n* = 12):** Calves with rapid fecal antigen test-confirmed infectious diarrhea that met at least two SIRS criteria, indicating clinical severity into a SIRS-positive systemic inflammatory state.
- **SIRS-Negative Group (*n* = 11):** Calves with rapid fecal antigen test-confirmed infectious diarrhea that met fewer than two SIRS criteria (presenting with zero or one criterion), representing localized enteric infection.
- **Healthy Control Group (*n* = 13):** Age-matched calves confirmed healthy by clinical and hematological evaluations, with negative rapid fecal antigen test results to exclude subclinical infection or carrier status.

Strict exclusion criteria were applied to standardize the study population and minimize potential confounding variables affecting serum biomarker concentrations. Calves were excluded if they had received antibiotics, nonsteroidal anti-inflammatory drugs (NSAIDs), corticosteroids, or vitamin-mineral supplements prior to clinical presentation. Additionally, calves with a history of dystocia or prematurity, or those presenting with concurrent infectious or systemic conditions (e.g., pneumonia, omphalitis), were excluded to ensure specificity of the observed systemic inflammatory response.

### 2.3. Blood Sample Collection and Hematological Analyses

Following clinical examination, blood samples were collected via a single venipuncture from the jugular vein of all calves in the study groups, strictly adhering to the principles of asepsis and antisepsis. For the diarrheic calves, this sampling was performed immediately upon admission to the clinic, prior to the administration of any fluid therapy or pharmacological intervention. Given the acute nature of neonatal calf diarrhea, the exact disease duration prior to admission typically ranged from 1 to 3 days. Furthermore, as clinical anorexia was prevalent among the patients, establishing a standardized fasting protocol (feeding status) was not clinically feasible or representative of the natural disease course. Blood sampling was performed into vacuum tubes containing K_2_ EDTA for hematological measurements, and into vacuum tubes with a serum separator gel for biochemical analyses. Complete blood count (CBC) evaluations were performed immediately from whole blood samples in EDTA tubes using a calibrated automated veterinary hematology analyzer (pocH-100i, Sysmex^®^, Kobe, Japan). These hematological analyses were conducted both to confirm the overall health status of the control group (ensuring all CBC parameters remained within normal physiological reference ranges) and to determine total white blood cell (WBC) counts—a fundamental component of the SIRS criteria—across all study groups. For ELISA analyses, blood samples collected in tubes with a clot activator and separator gel were allowed to stand at room temperature for 20–30 min for clot formation, and were subsequently centrifuged at 3000 rpm for 10 min. Serum samples were stored at −80 °C until analysis.

### 2.4. ELISA Measurements

Serum concentrations of Glucagon-Like Peptide-2 (GLP-2), Zonula Occludens-1 (ZO-1), occludin, and claudin-3—the primary biomarkers of the study—were measured using species-specific (bovine) commercial ELISA kits (BT Lab, Bioassay Technology Laboratory, Jiaxing, China; Cat. No.: E2333Bo, E2351Bo, E0483Bo, and E0370Bo, respectively). All washing, incubation, and optical reading procedures were carried out in strict accordance with the protocols provided by the manufacturers. The optical density values of the plates were determined using a standard microplate reader, and the concentrations were calculated based on the standard curves. To validate the analytical reliability of the obtained data, samples were analyzed in duplicate. All samples were measured simultaneously within a single analytical batch, and the optical densities were read at 450 nm. The intra-assay coefficients of variation were confirmed to be <8% for all assays. According to the manufacturer’s specifications, the detection limits were 3.47 ng/L for GLP-2, 0.20 ng/mL for ZO-1, 0.13 ng/mL for occludin, and 3.62 ng/L for claudin-3. Furthermore, the detection ranges for the assays were 7–1500 ng/L for GLP-2, 0.38–24 ng/mL for ZO-1, 0.25–16 ng/mL for occludin, and 7–2500 ng/L for claudin-3.

### 2.5. Statistical Analysis

Statistical analyses and data visualization were executed using SciPy (v1.12.0), statsmodels (v0.14.0), pandas, and seaborn packages within the Python programming environment (version 3.10). Prior to inferential testing, the distributional normality of continuous variables was assessed using the Shapiro–Wilk test, and homoscedasticity across groups was evaluated via Levene’s test.

For categorical variables, group comparisons were performed using Pearson’s chi-square test, whereas Fisher’s exact test was applied based on expected cell frequencies to evaluate the distribution of infectious diarrheal pathogens between SIRS-positive and SIRS-negative groups. Descriptive statistics for continuous variables were reported according to data distribution and variance homogeneity: normally distributed variables were expressed as mean ± standard deviation (SD), whereas non-normally distributed variables violating the normality assumption were presented as median (Q1–Q3 interquartile range).

Differences among the three independent study groups were analyzed using hypothesis tests tailored to data distribution. For variables satisfying parametric assumptions, a one-way ANOVA model was employed. When ANOVA indicated a statistically significant main effect, Tukey’s HSD post-hoc test was applied for pairwise comparisons. For multi-group comparisons of variables that violated parametric assumptions, the Kruskal–Wallis H test was utilized, followed by Dunn’s post-hoc test upon detecting significance. To control Type I error rate inflation inherent to multiple comparisons while preserving statistical power, *p*-values were adjusted using the Benjamini–Hochberg False Discovery Rate (FDR) procedure.

To delineate the strength and direction of linear or monotonic relationships between variables, correlation analyses were modeled separately for the SIRS-positive and SIRS-negative groups—rather than across the entire cohort—to preserve clinical specificity. Pearson’s correlation coefficient (*r*) was calculated when both paired variables were normally distributed, whereas Spearman’s rank correlation coefficient (rho) was employed when at least one variable deviated from normality. To assess the precision of these estimates within the relatively small sample size, 95% confidence intervals (CIs) for the correlation coefficients were calculated. Specifically, Fisher’s Z transformation was used for Pearson’s *r*, and a bootstrapping approach with 10,000 resamples was applied for Spearman’s rho. To prevent clutter within correlation matrices, only relationships with |*r*| ≥ 0.40 were considered, and strong associations with an absolute value above 0.50 that were statistically significant (*p* < 0.05) were modeled via scatter plots. To ensure a transparent presentation of individual data distributions, jittered points were integrated into all graphical representations. For all hypothesis tests, a two-tailed *p* < 0.05 was considered statistically significant.

## 3. Results

### 3.1. Demographic and Clinical Characteristics

Demographic and clinical characteristics of the 36 enrolled calves (12 SIRS-positive, 11 SIRS-negative, and 13 healthy controls) are presented in Table 1. No statistically significant differences were observed among groups regarding breed distribution, age, heart rate, or rectal temperature (*p* > 0.05). However, because the SIRS criteria for temperature and WBC encompass both subnormal and supranormal extremes, group means do not fully reflect individual clinical statuses. To ensure transparency in group classification, the number of calves fulfilling each specific SIRS criterion is detailed in Table 1. In contrast, respiratory rate differed significantly among groups (*p* = 0.0261), with post-hoc analysis demonstrating a significantly higher rate in the SIRS-positive group compared with healthy controls. Total white blood cell (WBC) counts also differed significantly among groups (*p* < 0.0001); post-hoc pairwise comparisons indicated that WBC levels were significantly higher in both the SIRS-positive and SIRS-negative groups than in healthy controls. Evaluation of enteropathogen distribution revealed that *Escherichia coli* K99 was the most prevalent pathogen across both diarrheic groups. Regarding the frequency of concurrent pathogens, single infections were identified in 9 calves and mixed infections in 3 calves within the SIRS-positive cohort. Similarly, the SIRS-negative cohort comprised 8 single and 3 mixed infections. Fisher’s exact test revealed no statistically significant differences in the detection rates of *E. coli* K99, Rotavirus, Coronavirus, or *Cryptosporidium* spp. between SIRS-positive and SIRS-negative calves (all *p* > 0.05; Table 1).

### 3.2. Biomarker Results

**Figure 1:**
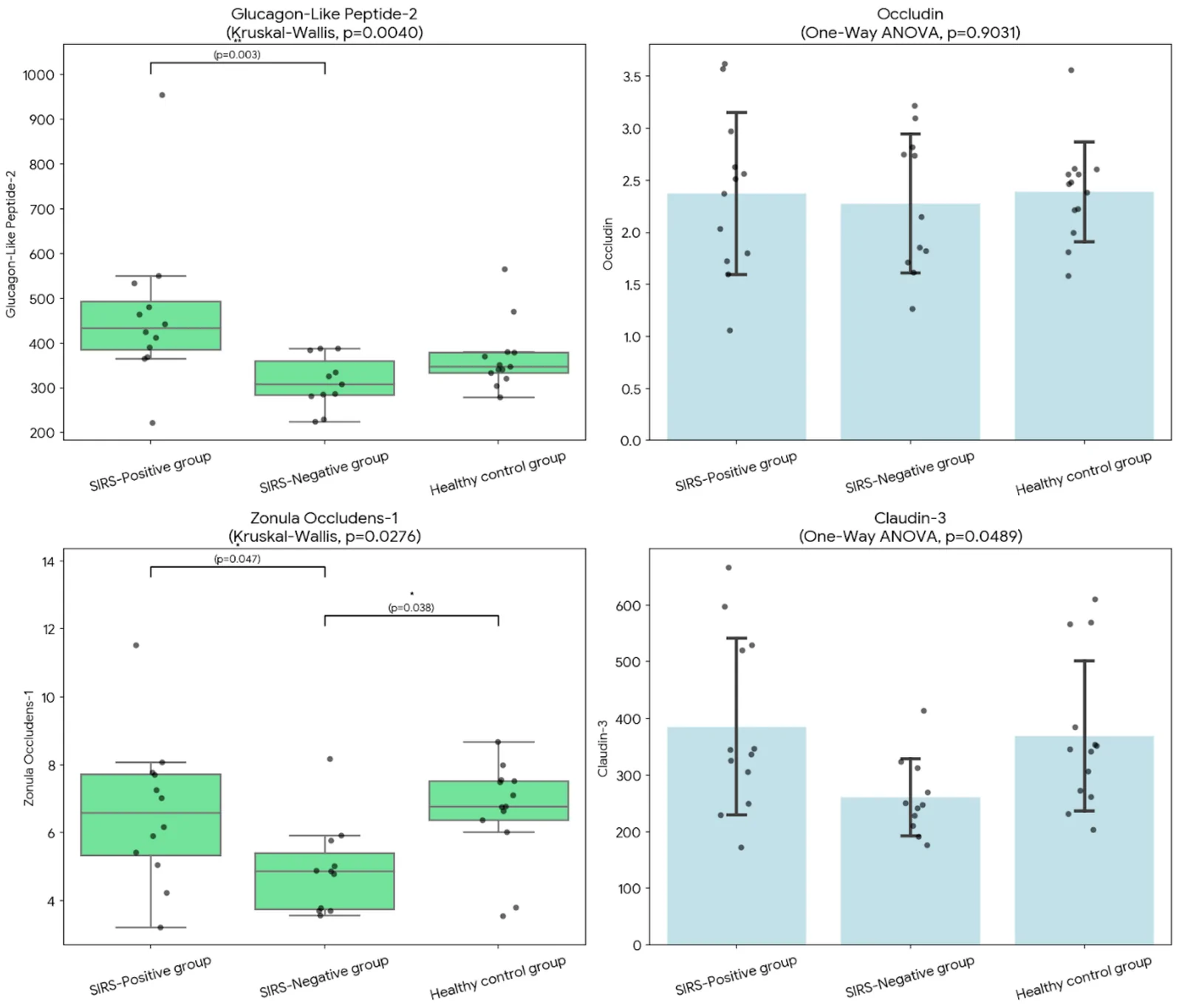
Comparison of serum Glucagon-Like Peptide-2 (GLP-2), Zonula Occludens-1 (ZO-1), Occludin, and Claudin-3 levels among healthy control, SIRS-negative, and SIRS-positive calf groups. Boxplots (for GLP-2 and ZO-1) display the median and interquartile range (Q1–Q3), while bar plots (for Occludin and Claudin-3) represent the mean and standard deviation. Jittered points represent the distribution of individual data. Overall significance was calculated using One-Way ANOVA for parametric data and the Kruskal–Wallis test for non-parametric data; statistically significant pairwise comparisons (post hoc) are indicated with *p*-values on the graphs. Asterisks denote statistically significant differences between the groups (* *p* < 0.05, ** *p* < 0.01). The dots represent the individual data points for each calf within the respective groups.

Serum biomarker concentrations and intergroup statistical comparisons are summarized in Figure 1 and Table 2. Serum GLP-2 levels differed significantly among groups (*p* = 0.0040); post-hoc analysis demonstrated that GLP-2 concentrations were significantly higher in SIRS-positive calves compared with SIRS-negative calves. Serum ZO-1 levels also varied significantly across groups (*p* = 0.0276), with post-hoc testing revealing significantly lower levels in the SIRS-negative group relative to both healthy controls and SIRS-positive calves. Although the omnibus analysis indicated a significant main effect for claudin-3 levels (*p* = 0.0489), post-hoc pairwise comparisons revealed no significant differences between individual groups. Serum occludin concentrations did not differ significantly among the study groups (*p* = 0.9031; Table 2).

### 3.3. Correlation Analyses

**Figure 2:**
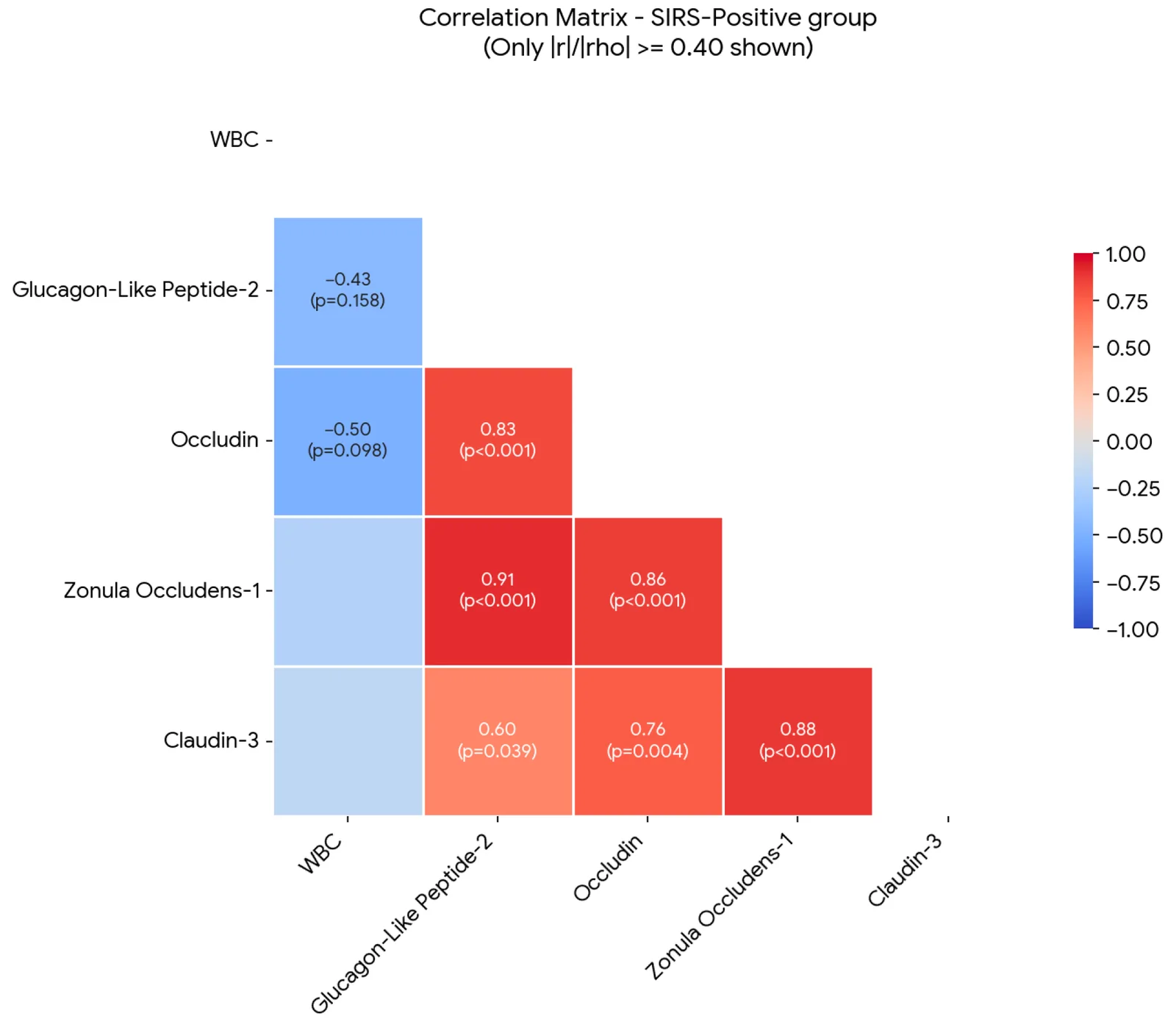
Correlation matrix (heatmap) illustrating the relationships between leukocyte (WBC) count, intestinal barrier proteins (ZO-1, Occludin, Claudin-3), and GLP-2 in the SIRS-positive group. To prevent visual clutter, only moderate to strong correlations (|*r*| or |rho| ≥ 0.40) are displayed. The color scale represents the strength and direction of the correlation coefficient, ranging from negative (blue) to positive (red).

**Figure 3:**
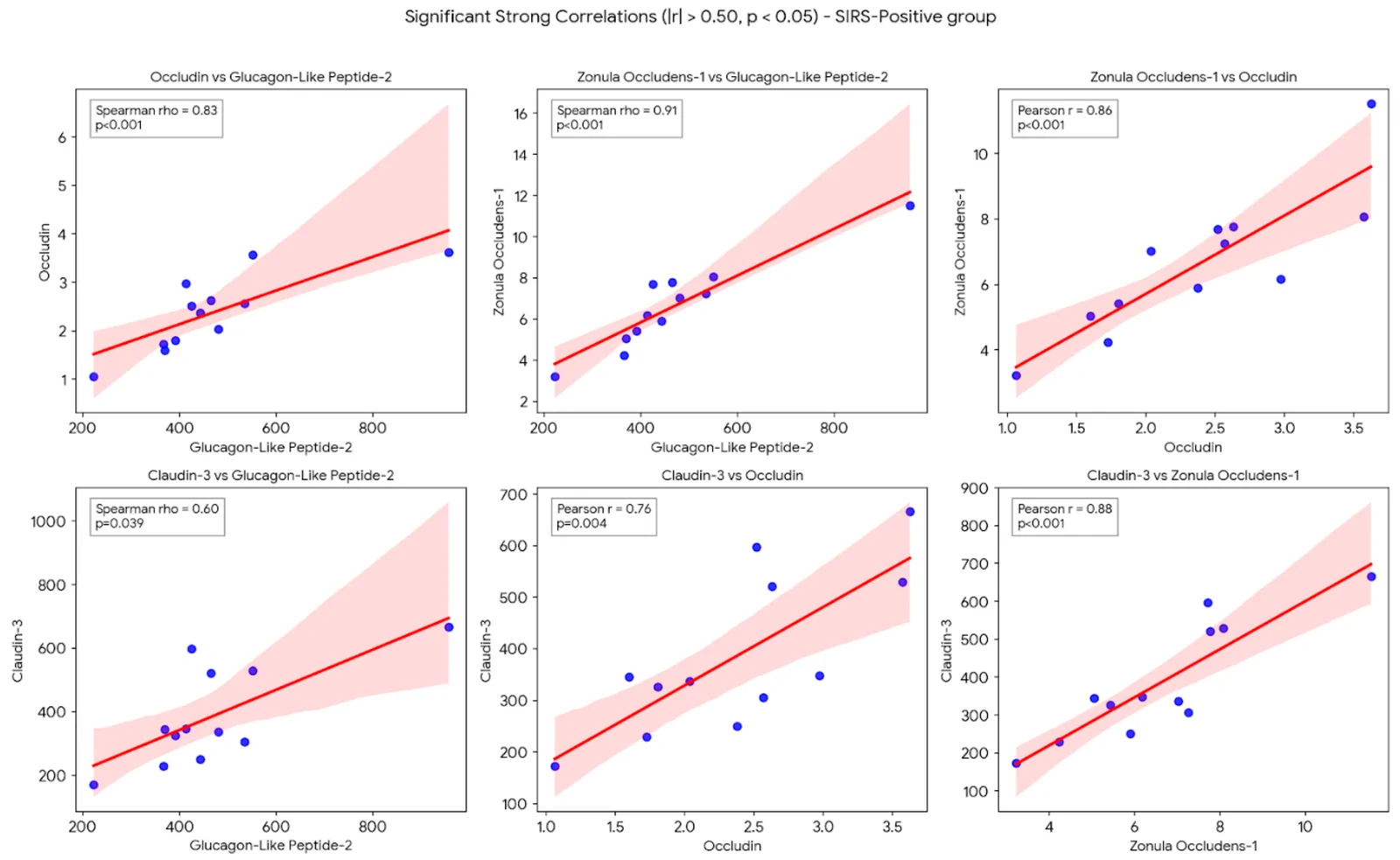
Scatter plots showing the significant and strong positive correlations (|*r*| > 0.50, *p* < 0.05) among GLP-2 and intestinal tight junction proteins in the SIRS-positive group. The red solid lines denote linear regression, and the pink shaded areas represent the 95% confidence intervals. The plots illustrate the strong positive correlations among intestinal tight junction proteins (ZO-1, Occludin, Claudin-3) and GLP-2 in the SIRS-positive group. The blue dots represent the individual data points for each calf.

**Figure 4:**
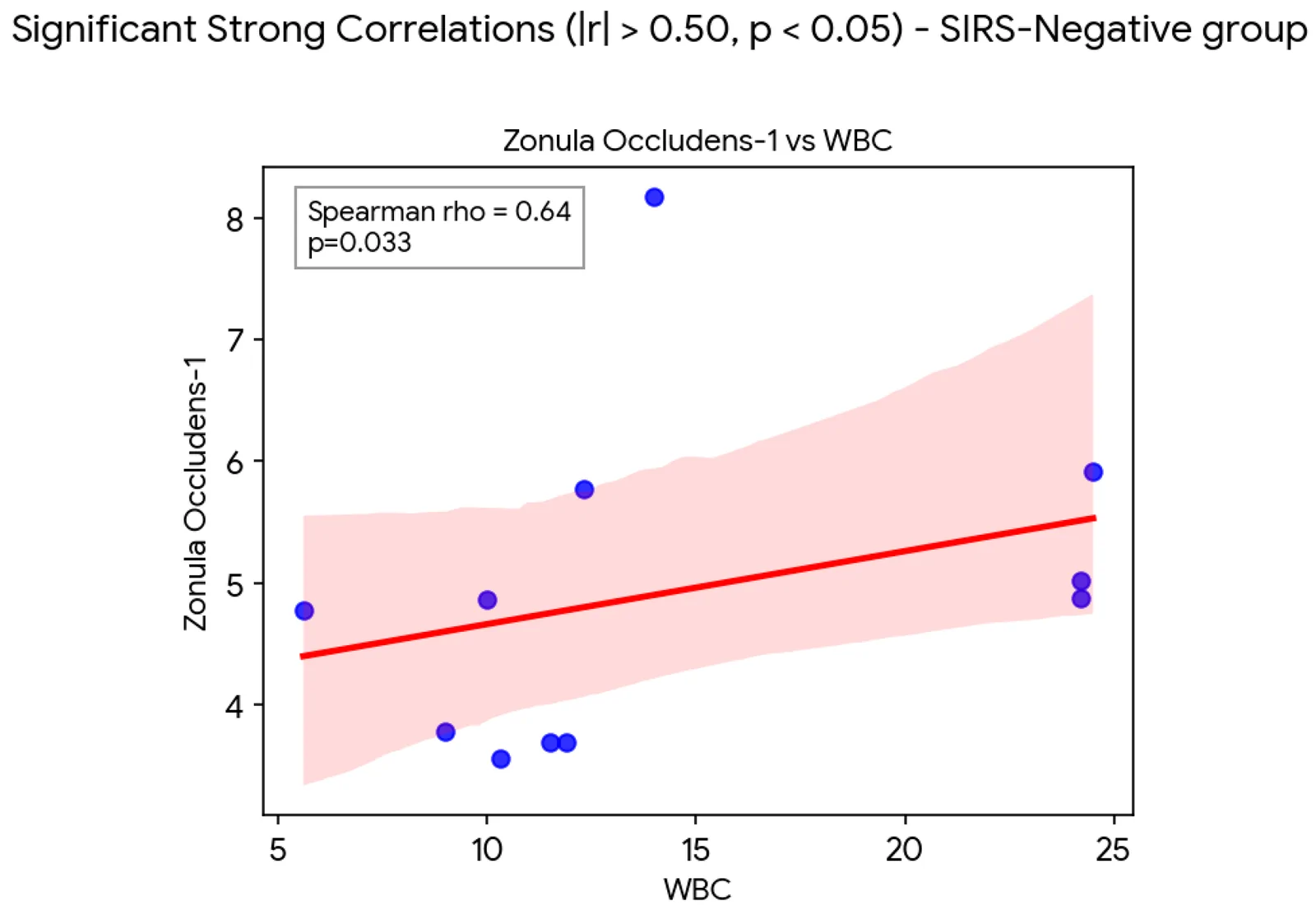
Scatter plot demonstrating the statistically significant and strong positive correlation (|*r*| > 0.50, *p* < 0.05) between Zonula Occludens-1 (ZO-1) and leukocyte (WBC) count in the SIRS-negative group. The red solid line indicates the linear regression trend, and the pink shaded area represents the 95% confidence interval (Spearman rho = 0.64, *p* = 0.033). The blue dots represent the individual data points for each calf.

The relational profiles among the study parameters were analyzed separately within the SIRS-positive and SIRS-negative groups. Among the coefficients (*r* or rho) calculated in the correlation matrices, variable pairs with an absolute value exceeding 0.50 and demonstrating statistical significance (*p* < 0.05) were modeled via correlation matrices and scatter plots. Accordingly, within the SIRS-positive group, statistically significant and strong correlations were identified between claudin-3 and ZO-1 (*r* = 0.88, 95% CI [0.62, 0.97]), claudin-3 and occludin (*r* = 0.76, 95% CI [0.33, 0.93]), claudin-3 and GLP-2 (rho = 0.60, 95% CI [−0.04, 0.95]), ZO-1 and occludin (*r* = 0.86, 95% CI [0.58, 0.96]), ZO-1 and GLP-2 (rho = 0.91, 95% CI [0.57, 1.00]), and occludin and GLP-2 (rho = 0.83, 95% CI [0.33, 0.99]) (Figure 2 and Figure 3). In contrast, within the SIRS-negative group, a statistically significant and strong correlation was detected exclusively between ZO-1 and WBC (rho = 0.64, 95% CI [0.11, 0.86]) (Figure 4).

## 4. Discussion

While the progression of neonatal calf diarrhea to a SIRS-positive state is known to involve intestinal barrier disruption [6,15,16], alterations in tight junction proteins [17,18], and GLP-2-mediated physiological responses [13], simultaneous clinical biomarker evaluations remain limited [19]. Accordingly, the present study sought to elucidate intestinal barrier profiles across varying levels of clinical severity. Our primary findings reveal that the SIRS-negative state is characterized by a significant reduction in serum ZO-1 levels. Conversely, in calves presenting with SIRS, circulating GLP-2 levels increase significantly while ZO-1 concentrations are maintained, suggesting a potential compensatory response mounted by the organism against increasing infection severity.

In neonatal calf diarrhea, the transition from a localized enteric infection to a systemic inflammatory response is characterized by specific clinical and hematological shifts. In the present study, the cohorts were stratified based on these established parameters to accurately reflect distinct physiological states. For instance, the elevated WBC counts utilized as a baseline inclusion criterion for the diarrheic groups reflect the development of a localized or systemic cellular immune response, which is consistent with data reported in the veterinary literature [20,21,22]. Furthermore, the inclusion criteria for the severe clinical cohort inherently captured animals exhibiting tachypnea; in the literature, tachypnea and acidemia are recognized as primary clinical indicators of a circulatory compensatory effort during systemic inflammation [23,24]. Consequently, structuring the study groups around these validated clinical SIRS criteria ensures that the biomarker evaluations are grounded in well-defined physiological foundations.

In the SIRS-negative group, which represents the early stage of localized enteric infections, levels of ZO-1—a core protein of the intestinal barrier—were found to be significantly lower than those in both the healthy control and SIRS-positive groups. This decline observed in a phase where a SIRS-positive condition has not yet developed may reflect the direct structural damage exerted by specific diarrheal pathogens within the intestinal lumen. Indeed, cell-based studies have reported that pathogenic microorganisms and toxins disrupt localized epithelial intracellular signaling pathways prior to inducing systemic shock, thereby increasing intestinal permeability by dissociating tight junction proteins such as ZO-1 from the cell membrane [10,25,26]. Consequently, our finding in the SIRS-negative group is considered to reflect the localized disruption of the intestinal mechanical barrier occurring during the early phase of infection. However, it is important to discuss the limitations of interpreting circulating ZO-1 concentrations as a direct indicator of intestinal barrier integrity. Because serum ZO-1 represents an indirect proxy rather than a direct histological evaluation, its definitive diagnostic reliability remains a debated topic [27]. This debate primarily stems from the widespread expression of tight junction proteins; ZO-1 is also present in non-intestinal tissues, such as the vascular endothelium, meaning systemic inflammation could theoretically contribute to the circulating pool [28]. Moreover, as clinical severity increases, maintained or elevated serum concentrations may not necessarily indicate intact tissue expression or active barrier repair; rather, they could reflect the extensive release of these structural proteins into the bloodstream following severe epithelial injury. Furthermore, the correlation between peak serum ZO-1 levels and the exact severity of mucosal histological damage is not yet fully standardized. Therefore, while the current findings offer a preliminary perspective on these pathophysiological profiles, serum ZO-1 should be evaluated as an adjunct biomarker rather than an absolute diagnostic measure of intestinal permeability.

Despite these diagnostic limitations, statistical analyses revealed a strong positive correlation between ZO-1 levels and WBC counts within the SIRS-negative group. It is well established that the process of intestinal barrier injury is not merely a structural dissolution but progresses concurrently with the local immune response [29]. Increased intestinal permeability and the breakdown of tight junction protein (TJP) complexes can function as a signaling mechanism that triggers leukocyte infiltration into the region [30,31]. In this context, the interrelated data between ZO-1 alterations and WBC counts obtained in our study offer a supporting perspective for understanding cellular interactions within disease mechanisms.

Conversely, in the SIRS-positive cohort, representing a more severe clinical stage, the physiological landscape was found to shift in a different direction. Although a more severe structural injury to the intestinal barrier would be expected under conditions of advancing infection and systemic shock, the preservation of ZO-1 levels in the SIRS-positive group at healthy animal levels, accompanied by a significant increase in GLP-2 levels compared to the SIRS-negative group, points to a potential concurrent physiological response mounted by the organism. The existing literature indicates that conditions such as severe systemic inflammation or endotoxemia trigger GLP-2 upregulation as a protective reflex within the organism [14,32]. Given the functions of GLP-2 as an intestine-specific growth factor, we hypothesize that the elevated GLP-2 levels detected in our SIRS-positive cases might represent an exploratory physiological compensatory response associated with clinical severity, rather than direct evidence of active mucosal repair. Consequently, the preservation of ZO-1 levels observed in the SIRS-positive group could theoretically be associated with this GLP-2 pathway. However, as emphasized previously, maintained circulating ZO-1 levels could also reflect extensive release of these structural proteins into the bloodstream following severe epithelial injury, rather than active tissue repair. Because our current data are limited to associations between circulating biomarkers, further experimental evidence is required to definitively determine whether this preservation is actively mediated by a GLP-2-driven tissue repair mechanism.

This compensation hypothesis is further supported at the cellular level by the positive correlation findings observed among GLP-2, ZO-1, occludin, and claudin-3 within the SIRS-positive group. Our data demonstrate that these proteins, which exhibit more independent profiles in a healthy organism, display a highly coordinated relationship with GLP-2 during systemic inflammation. It has been reported that during severe inflammatory states such as sepsis, barrier proteins do not completely disappear; instead, the fluctuations observed in circulation may reflect coordinated biomarker changes rather than directly evaluated structural tissue remodeling [33,34]. Although no significant intergroup differences were detected in claudin-3 and occludin levels in our study, the linear relationship these markers exhibited with GLP-2 during the severe clinical phase is considered an exploratory indicator of their concurrent involvement in the physiological response. However, given the relatively small sample size, these correlational findings should be interpreted cautiously as exploratory and hypothesis-generating rather than definitive mechanistic proof, thereby mitigating the risk of overinterpretation.

Furthermore, evaluating the correlations between the measured variables provides an exploratory perspective on how these biomarkers associate across different levels of clinical severity. In the SIRS-negative group, the positive correlation between serum ZO-1 and total leukocyte counts may reflect tight junction protein release into the bloodstream concurrent with initial immune mobilization. When evaluating calves with a more severe clinical presentation (the SIRS-positive group), significant positive correlations were observed between GLP-2 and tight junction proteins. Given the cross-sectional design of this study, these statistical associations cannot establish causality, temporal disease progression, or active tissue remodeling. Nevertheless, they highlight that as the clinical picture worsens into a systemic state, the organism exhibits a concurrent physiological response. Whether this coordinated fluctuation in circulating biomarkers reflects a compensatory host mechanism against systemic inflammation or merely mirrors the extent of epithelial injury remains an important area for future longitudinal investigation.

The limitations of this study warrant careful consideration. First, the cross-sectional design inherently precludes establishing definitive causality, and the sample size—particularly within specific subgroups—remains relatively small for correlation analyses. Second, structural changes in intestinal permeability were evaluated indirectly via circulating biomarkers rather than gold-standard methods, such as tissue biopsy or functional permeability assays. Third, the lack of microbiological confirmation (e.g., blood cultures) meant that the classification of systemic involvement relied on clinical, hematological and SIRS criteria, as detailed blood gas and perfusion indices were not systematically recorded. Furthermore, while rapid antigen screening provided initial diagnostic utility, these assays detect pathogen shedding rather than establishing primary etiological causality, leaving potential asymptomatic carriage or undetected co-infections unaccounted for. Crucially, as rapid antigen negativity cannot definitively exclude all subclinical infections, the presence of low-grade asymptomatic carriage in the healthy control group remains a possibility. Although the main objective of this study was to evaluate general host physiological responses rather than pathogen-specific profiles, these diagnostic boundaries should be taken into account when interpreting biomarker patterns. Nevertheless, obtaining these preliminary data via a minimally invasive approach under field conditions provides a practical basis for future mechanistic investigations.

## 5. Conclusions

In conclusion, the present findings suggest that circulating GLP-2 and ZO-1 parameters may reflect cellular-level intestinal barrier injury and subsequent alterations in permeability in diarrheic neonatal calves. Given the cross-sectional nature of the data, the coordinated fluctuations between GLP-2 and tight junction proteins in SIRS-positive calves should be interpreted as exploratory associations rather than definitive evidence of mediated barrier repair. These markers represent promising candidate biomarkers requiring validation in larger prospective studies to establish their clinical utility for evaluating clinical severity from localized enteric infection to a SIRS-positive systemic inflammatory state and for evaluating physiological compensatory responses.

## Figures and Tables

**Table 1:** Demographic, clinical, and hematological findings, and the distribution of identified infectious agents among the SIRS-positive, SIRS-negative, and healthy control calf groups.

Variable	SIRS-Positive Group (*n* = 12)	SIRS-Negative Group (*n* = 11)	Healthy Control Group (*n* = 13)	*p*-Value	Post Hoc
Age (Day)	9.00 ± 3.98	11.27 ± 5.55	11.00 ± 5.87	0.5169	N/A
WBC	17.80 (15.50–19.40)	11.90 (10.15–19.10)	7.50 (6.24–9.36)	<0.0001	SIRS-positive > Healthy control; SIRS-negative > Healthy control
Pulse	121.08 ± 33.80	114.09 ± 15.26	126.69 ± 14.10	0.4148	N/A
Respiration	48.17 ± 21.75	38.64 ± 6.05	32.77 ± 6.34	0.0261	SIRS-positive > Healthy control
Rectal Temperature	38.11 ± 1.70	38.56 ± 0.51	38.96 ± 0.40	0.1422	N/A
Distribution of fulfilled SIRS criteria, *n* (%)					
Abnormal Body Temperature	8 (66.7%)	4 (36.4%)			
Tachycardia	7 (58.3%)	3 (27.3%)			
Tachypnea	7 (58.3%)	7 (63.6%)			
Abnormal WBC	12 (100%)	5 (45.5%)			
Infectious Agents, *n* (%)					
*E. coli* K99	8 (66.7%)	6 (54.5%)		0.6802	N/A
*Rota* virus	5 (41.7%)	4 (36.4%)		1.000	N/A
*Corona* virus	4 (33.3%)	5 (45.5%)		0.6802	N/A
*Cryptosporidium* spp.	1 (8.3%)	4 (36.4%)		0.1550	N/A

Abbreviations: WBC, white blood cell; N/A, not assessed.

**Table 2:** Comparison of serum Glucagon-Like Peptide-2 (GLP-2) and intestinal tight junction protein (Occludin, Zonula Occludens-1, Claudin-3) concentrations among the SIRS-positive, SIRS-negative, and healthy control calf groups.

Parameters	SIRS-Positive Group (*n* = 12)	SIRS-Negative Group (*n* = 11)	Healthy Control Group (*n* = 13)	*p*-Value	Post Hoc
GLP-2 (ng/L)	433.90 (385.21–493.68)	308.75 (283.63–359.72)	347.42 (333.66–379.65)	0.0040	SIRS-negative vs. SIRS-positive
Occludin (ng/mL)	2.37 ± 0.78	2.28 ± 0.67	2.39 ± 0.48	0.9031	N/A
Zonula Occludens-1 (ng/mL)	6.60 (5.33–7.72)	4.87 (3.74–5.40)	6.78 (6.38–7.52)	0.0276	SIRS-negative vs. SIRS-positive; Healthy control vs. SIRS-negative
Claudin-3 (ng/L)	385.66 ± 155.61	260.88 ± 68.06	369.46 ± 132.47	0.0489	No significant difference detected

Abbreviations: GLP-2, glucagon-like peptide-2; ZO-1, zonula occludens-1; N/A, not assessed.

## Data Availability

The data presented in this study are available on request from the corresponding author due to privacy and confidentiality restrictions regarding the animal owners.
